# Efficient Entropic Security with Joint Compression and Encryption Approach Based on Compressed Sensing with Multiple Chaotic Systems

**DOI:** 10.3390/e24070885

**Published:** 2022-06-27

**Authors:** Jingya Wang, Xianhua Song, Ahmed A. Abd El-Latif

**Affiliations:** 1 School of Science, Harbin University of Science and Technology, Harbin 150080, China; 2020900017@stu.hrbust.edu.cn; 2 EIAS Data Science Lab, College of Computer and Information Sciences, Prince Sultan University, Riyadh 11586, Saudi Arabia; 3 Department of Mathematics and Computer Science, Faculty of Science, Menoufia University, Shebin El-Koom 32511, Egypt

**Keywords:** image encryption, compressed sensing, chaotic system, bit-cycle operation, double XOR operation

## Abstract

This paper puts forward a new algorithm that utilizes compressed sensing and two chaotic systems to complete image compression and encryption concurrently. First, the hash function was utilized to obtain the initial parameters of two chaotic maps, which were the 2D-SLIM and 2D-SCLMS maps, respectively. Second, a sparse coefficient matrix was transformed from the plain image through discrete wavelet transform. In addition, one of the chaotic sequences created by 2D-SCLMS system performed pixel transformation on the sparse coefficient matrix. The other chaotic sequences created by 2D-SLIM were utilized to generate a measurement matrix and perform compressed sensing operations. Subsequently, the matrix rotation was combined with row scrambling and column scrambling, respectively. Finally, the bit-cycle operation and the matrix double XOR were implemented to acquire the ciphertext image. Simulation experiment analysis showed that the compressed encryption scheme has advantages in compression performance, key space, and sensitivity, and is resistant to statistical attacks, violent attacks, and noise attacks.

## 1. Introduction

In the wake of the development of the internetworking and information technique, digital images are extensively used in numerous domains [1,2,3,4]. A great quantity of information is presented in a digital image form, which usually contains private and important information. When important information is falsified or leaked, it can cause acute consequences [5,6], which makes the privacy security issue very prominent. Hence, the information security protection of digital images has aroused widespread attention [7,8]. In this situation, multiple encryption scenarios have emerged.

In the past few years, due to the excellent characteristics of chaotic maps [9,10], various encryption scenarios based on chaotic maps have been created [11,12,13,14]. Wang et al. utilized parameter controlled scroll chaotic attractors for encryption [15]. Gao proposed a new 2D hyperchaotic system for image encryption [16]. In addition, chaotic maps can be combined with a variety of methods for encryption. Chen et al. combined chaotic maps and DNA coding for encryption and the results indicated that the effect was better than using chaotic maps alone [17,18]. Yu et al. combined chaotic maps and fractional Fourier transform for optical image encryption [19,20]. Choi et al. combined chaotic maps and cellular automata for encryption [21,22]. Sundarakrishnan et al. used chaotic mapping and cellular automata to encrypt color images, increased the key space, and used a double permutation and replacement framework, which significantly reduced the correlation and improved the security of the algorithm [23]. Based on the advantage of chaos theory to encryption, many scholars began to use multiple chaotic systems in the encryption framework. Ramasamy et al. achieved secure and efficient encryption using the proposed enhanced logical map, chaotic map, and general encryption framework—scrambling, diffusing, and generating a key stream [24]. Masood et al. used multiple chaotic systems such as two-dimensional Arnold cat mapping, Newton–Leipnik dynamic system and improved Logistic–Gaussian chaotic system, to generate sequences for multiple links of color image encryption, which improved the security of the algorithm [25]. This image encryption scheme using multiple chaotic systems combined with the encryption framework makes full use of the advantages of chaos for encryption, making encryption more secure and efficient, obtaining good encryption effects under various experimental tests, and resisting various attacks. Although the above-mentioned algorithms have achieved good results, none of the above algorithms are applicable due to the bandwidth constraint problem.

To satisfy the bandwidth-constrained demands, the theoretical concept of compressed sensing (CS) was established [26,27]. Soon afterward, multifarious compressed encryption scenarios based on CS were put forward [28,29]. Lu et al. created an image encryption scenario [30] that compressed images by CS and encrypted images by double random phase coding technology. Although this algorithm reduced the amount of data, its method of using the metric matrix as the key takes up a large amount of storage space and is bandwidth-constrained.

To resolve these issues, a new image compression encryption scenario has attracted much attention [31,32,33,34,35,36]. This scenario combines compressed sensing with chaotic systems, which utilizes compressed sensing to compress the image to meet the bandwidth demands in transmission and can also make full use of the excellent properties of the chaotic system by using the initial parameters of chaotic maps as the key, and using the created sequences to form the measurement matrix. This method resolves the problem that the key occupies a large storage space and the limited bandwidth.

To further improve the security of these schemes, many scenarios have adopted scrambling methods [37,38,39,40,41]. According to the position of the scrambling in the algorithm, these can be divided into two categories. One is to perform the scrambling and confusion operation after the measurement value is obtained by compressed sensing [40,41]. The other is to first obtain a sparse coefficient matrix through a sparse transformation of the original image and then perform a scrambling operation on the sparse coefficient matrix [38,39]. Both types of methods can decrease the image correlation and heighten the security of the scenarios, while the latter has the advantage of effectively enhancing the reconstruction quality of the decrypted image [42]. In general, there are two scrambling methods in the encryption process: scrambling using Arnold map [38] and scrambling of the index values obtained by sorting the chaotic sequences [37,38]. Both methods have drawbacks. The Arnold map scrambling method cannot be directly used for non-square images [43]. The second scrambling method is easy to operate and its scrambling effect is determined by the randomness of the chaotic sequence [44], so it is not suitable for a chaotic system with bad randomness. Therefore, a scrambling method called pixel transformation is proposed.

To increase the security and meet the demand of limited bandwidth, a compressed encryption plan based on two chaotic systems and CS was put forward in this paper. First, the SHA-384 of the original image was used to calculate the initial parameters of the 2D-SLIM and 2D-SCLMS system and used as the key, which greatly heightened the relevance between the scheme and the plaintext, and can better resist known plaintext attacks and selective plaintext attacks. Second, the plaintext image is converted into a sparse coefficient matrix. Third, to increase the reconstruction quality of the decrypted image, a new scrambling technology is created. In addition, the chaotic sequence is utilized to create the measurement matrix and implement the compressed sensing operation, which greatly meets the transmission bandwidth requirements. To further heighten the security, the encryption operation combines matrix rotation with row scrambling and column scrambling, respectively, followed by a bit-cycle operation. Finally, double XOR of the matrix is implemented to acquire the ciphertext image.

The novelties of this paper are: (1) By combining two chaotic systems and compressed sensing, a new image encryption scheme is generated; (2) a new pixel transformation scrambling method is proposed; and (3) the combination of matrix rotation and scrambling improves the security of the algorithm.

The remaining sections are organized as follows. Section 2 presents the related work. Section 3 designs the new compression encryption scenario. Section 4 demonstrates the corresponding decryption algorithms. Section 5 presents the various performance analyses of the compression encryption scenario. Section 6 provides our conclusions.

## 2. Related Works

### 2.1. Compressed Sensing

In 2006, Donoho et al. proposed a compressed sensing formulation and processing method for signals [26,27]. This concept smashes the restrictions of Shannon’s sampling theorem by exploiting the sparsity of the natural signal itself or the sparsity of a certain transform domain, allowing for the recovery of the sampled signal with a small amount of samples at lower than the Nyquist sampling rate. Compressed sensing, also known as compressive sampling, allows for sampling, compression, and encryption to be conducted concurrently [43,45].

The pivotal elements of compressed sensing comprises sparse representation, the measurement matrix, and the reconstruction scheme. In general, the signal is not sparse in the time domain, but in some transform domains, the signal may become sparse. Therefore, the classic sparsity representation methods comprise discrete wavelet transform (DWT), fast Fourier transform (FFT), and discrete cosine transform (DCT).

We took a 1D signal to explain the step of compressed sensing. The sparsity expression for a non-sparse signal *x* (*N* × 1) in the transform domain is

(1) x=Ψs

In Equation (1), Ψ (*N* × *N*) is known as the normal orthogonal matrix and *s* (*N* × 1) is a *K* sparse vector.

According to Equation (1), the specific expression of compressed sensing is

(2) y=Φx=ΦΨs=Θs

In Equation (2), Φ (*M* × *N*) is the measurement matrix; Θ (*M* × *N*) is the sensing matrix; and *y* (*M* × 1) is the measured value matrix. In particular, *M* < *N*.

Compressed sensing demands that Θ has the content of the restricted isometry property [46], that is to say, Φ and Ψ are uncorrelated. In addition, the length of *y* ought to be

(3) $$M\geq cK\log\frac{N}{K}$$

In Equation (3), *c* is a constant with a small value.

To exactly recover the *s* from the measured value matrix *y*, theoretically, the problem of *l*_0_ norm minimization should be solved

(4) $$\begin{array}{l} \min||s|{|}_{0}\\ s.t.y=\Phi \Psi s \end{array}$$

However, Equation (4) is an *NP*-hard problem. Therefore, in general, the problem of *l*_1_ norm minimization is used to supersede Equation (4)

(5) $$\begin{array}{l} \min||s|{|}_{1}\\ s.t.y=\Phi \Psi s \end{array}$$

There are many reconstruction algorithms for compressed sensing, the most common ones are the orthogonal matching tracking algorithm, subspace pursuit algorithm, and the smooth *l*_0_ norm (*Sl*_0_) algorithm. We chose the *Sl*_0_ algorithm for the reconstruction in this paper.

### 2.2. Sigmoid Function

A common S-shaped function, also known as an S-shaped growth curve, is the Sigmoid function [39], whose expression is

(6) $$y=\frac{a}{1+e^{-b(x-c)}}$$

where the range of *y* is [0, *a*]. We utilized the sigmoid function for quantization, so we set *a* = 255, *b* = 80/(15.518 × (*X*_max_ − *X*_min_)), *c* = (*X*_max_ + *X*_min_)/2. *X*_max_ and *X*_min_ are the maximum and minimum values of *X*, respectively. For different images, *X*_max_ and *X*_min_ are different, (i.e., the values of *b* and *c* are taken differently).

### 2.3. Chaotic System

#### 2.3.1. 2D-SCLMS System

The 2D-SCLMS map is a hyperchaotic system generated based on Logistic and Sine maps [47] with the expression

(7) $$\begin{array}{l} x_{i+1}=\sin(4\pi^{2}(\mu \sin(4\pi x_{i}(1-x_{i})))+4uy_{i}(1-y_{i}))\\ y_{i+1}=\sin(4\pi^{2}(\mu \sin(4\pi y_{i}(1-y_{i})))+4ux_{i+1}(1-x_{i+1})) \end{array}$$

where *μ* > 0.1 is the parameter. *x_i_*, *y_i_*∈ (−1,1), *i* = 1, 2,….

#### 2.3.2. 2D-SLIM

The 2D-SLIM is a chaotic map with complex properties for image encryption [48]. Its expression is

(8) $$\begin{array}{l} x_{i+1}=\sin(\mu_{1}y_{i})\sin(50/x_{i})\\ y_{i+1}=\mu_{2}(1-2x_{i+1}^{2})\sin(50/y_{i}) \end{array}$$

where *μ*_1_, *μ*_2_ ∈ (0,+∞), *x_i_*, *y_i_* ∈ (−1,1), *i* = 1, 2, … We set *μ*_1_ = 2π, *μ*_2_ = 1.

## 3. Image Encryption Process

**Figure 1:**
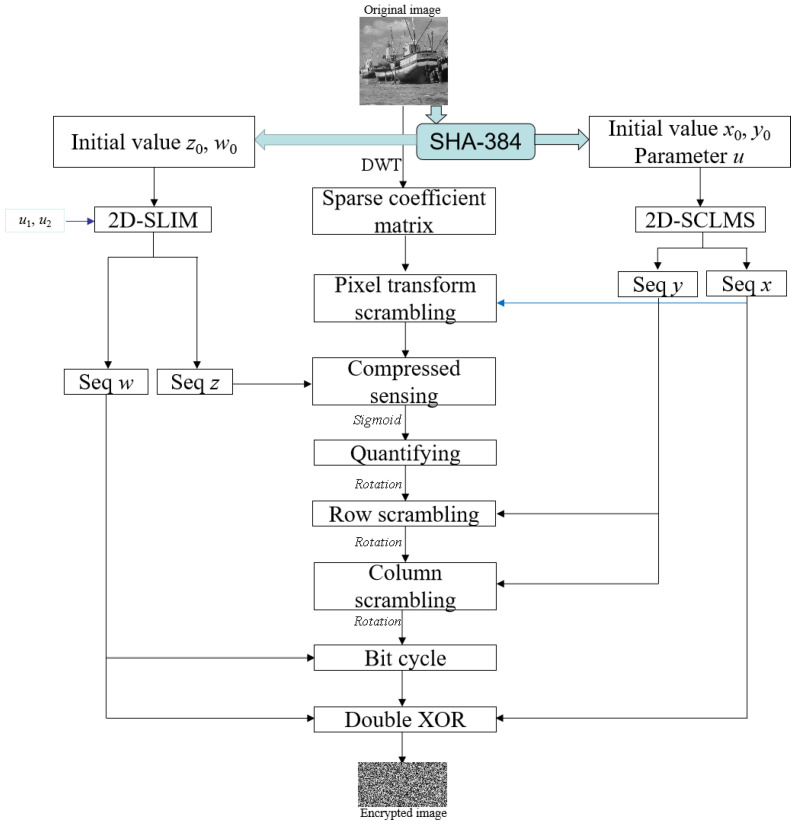
The flow chart of the proposed encryption algorithm.

A new encryption scenario was created and its flow chart is presented in Figure 1.

### 3.1. Key Generation

The hash algorithm was utilized to create the initial parameters of the 2D-SCLMS map and the 2D-SLIM, which enhanced the relevance between the ciphertext image and the original image. First, the SHA-384 hash function generates a binary sequence composed of 384 bits and then this sequence is separated into blocks every 8 bits (i.e., 48 decimal numbers *h*_1_, *h*_2_, …, *h*_48_). The 2D-SCLMS system is mainly used for pixel transformation, row scrambling, and column scrambling, and the initial values and parameters are calculated as

(9) $$\begin{array}{l} x_{0}=\mathrm{mod}(\sum_{i=1}^{10}k_{i},256)/256\\ y_{0}=\mathrm{mod}(\sum_{i=11}^{20}k_{i},256)/256\\ u=\mathrm{mod}(\sum_{i=21}^{30}k_{i},256)/256+\alpha \end{array}$$

The 2D-SLIM is mainly utilized to establish the measurement matrix and perform bit-cycle, where the initial values are calculated as

(10) $$\begin{array}{c} a=\mathrm{mod}(\sum_{i=43}^{48}k_{i},256)/2560\\ \;z_{0}=\mathrm{mod}(\sum_{i=31}^{36}k_{i},256)/256+a\\ \;w_{0}=\mathrm{mod}(\sum_{i=37}^{42}k_{i},256)/256 \end{array}$$

### 3.2. Encryption Process

The proposed encryption algorithm is depicted as follows.

**Step 1:** The initial parameters (*x*_0_, *y*_0_, *u*), obtained in Section 3.1, are entered into the 2D-SCLMS map for 500 + *N*^2^ iterations. The first 500 values are removed to acquire the sequences *X*, *Y*. Sequence *X*_1_ is obtained

(11) X1=mod(round(X×10^8),4)

*X*_1_ is divided equally into four sequences and each sequence is transformed into an *N*/2 × *N*/2 matrix named *X*_11_, *X*_12_, *X*_13_, *X*_14_.

The sequence *X* is transformed into a matrix *X*_2_ (*N* × *N*) and *X*_2_ is divided into *X*_2__1_, *X*_2__2_ by rows, so the matrices *A*, *B* are obtained, respectively.

(12) $$\begin{array}{c} X_{21}=X_{2}(1:N\times CR,:)\\ X_{22}=X_{2}((1-CR)\times N+1:end,:)\\ A=mod(floor(X_{21}\times 10^{10}),256)\\ B=mod(floor(X_{22}\times 10^{10}),256) \end{array}$$

The sequence *Y* is transformed into an *N* × *N* matrix and is then divided into two parts *Y*_1_, *Y*_2_, according to the number of rows. The matrix *Y*_1_ is arrayed in descending order by the columns, and the matrix *Y*_2_ is arrayed in ascending order by rows to obtain the index matrix *L*_1_, *L*_2_, respectively.

(13) $$\begin{array}{c} Y_{1}=Y(1:N\times CR,:)\\ Y_{2}=Y((1-CR)\times N+1:end,:)\\ \left[\sim ,L_{1}]=sort(Y_{1},2,‘descend’\right)\\ \left[\sim ,L_{2}]=sort(Y_{2}\right) \end{array}$$

**Step 2:** The plaintext image *P* (*N* × *N*) generates a discrete coefficient matrix *P*_1_ through DWT, and then matrix *P*_1_ is divided equally into four small matrices *P*_11_, *P*_12_, *P*_13_, and *P*_14_.

(14) $$P_{1}=\Psi \times P\times \Psi^{T}$$

**Step 3:** Perform pixel transformation on *P*_11_, *P*_12_, *P*_13_, *P*_14_ using matrices *X*_11_, *X*_12_, *X*_13_, *X*_14_. Take *X*_11_ as an example for illustration.

(15) $$\mathrm{If}X_{11}(i,j)=0,\mathrm{then}\;\mathrm{temp}=P_{11}(i,j)\;P_{11}(i,j)=P_{12}(i,j)\;P_{12}(i,j)=\mathrm{temp}\;\mathrm{If}X_{11}(i,j)=1,\mathrm{then}\;\mathrm{temp}=P_{11}(i,j)\;P_{11}(i,j)=P_{13}(i,j)\;P_{13}(i,j)=\mathrm{temp}\;\mathrm{If}X_{11}(i,j)=2,\mathrm{then}\;\mathrm{temp}=P_{11}(i,j)\;P_{11}(i,j)=P_{14}(i,j)\;P_{14}(i,j)=\mathrm{temp}\;\mathrm{If}X_{11}(i,j)=3,\mathrm{then}\;\mathrm{temp}=P_{11}(i,j)\;P_{11}(i,j)=P_{11}(\frac{N}{2}+1-i,\frac{N}{2}+1-j)\;P_{11}(\frac{N}{2}+1-i,\frac{N}{2}+1-j)=\mathrm{temp}$$

Similarly, pixel transformation was performed again based on the values of *X*_12_, *X*_13_, *X*_14_, respectively. When the pixel transformation was over, the four matrices were combined to acquire *P*_2_.

**Step 4:** The initial values (*z*_0_, *w*_0_), created in Section 3.1 and the parameters, are entered into the 2D-SLIM iterating 500 + *d* × *M* × *N* times to produce two chaotic sequences. The first 500 values of the two sequences are removed to obtain the chaotic sequence *Z*, *W*. *M* = CR × *N*, wherein CR is the compression rate and *d* is the sampling distance.

Sequence *Z*_1_ is acquired by sampling from sequence *Z* according to the sampling distance *d*. The measurement matrix Φ (*M* × *N*) is generated.

(16) $$\begin{array}{c} Z_{i}^{\prime }=1-2Z_{1+id},i=1,2,\cdots ,MN\\ \Phi =\sqrt{\frac{2}{M}}reshape(Z_{i}^{\prime },M,N) \end{array}$$

Take the *MN* values from the sequence *W* and transform it into a matrix *W*_1_. According to Equation (17), *W*_2_ and *C* can be generated.

(17) $$\begin{array}{c} W_{2}=\mathrm{mod}(floor(W_{1}\times 10^{6}),8)\\ C=\mathrm{mod}(floor(W_{1}\times 10^{6}),256) \end{array}$$

**Step 5:** Compress *P*_2_ to obtain the measurement results *P*_3_.

(18) $$P_{3}=\Phi \times P_{2}$$

**Step 6:** Quantize *P*_3_ according to the sigmoid function introduced in Section 2.2 and round the quantized result to obtain *P*_4_.

(19) $$P_{4}=\frac{a}{1+e^{-b(P_{3}-c)}}$$

**Step 7:** Rotate *P*_4_ counterclockwise by 180° and then scramble the columns according to the index matrix *L*_1_ to obtain *P*_5_.

(20) $$\begin{array}{c} P_{41}=rot90(rot90(P_{4}))\\ P_{5}(i,j)=P_{41}(i,L_{1}(i,j)) \end{array}$$

**Step 8:** Rotate *P*_5_ counterclockwise by 180° and then scramble the rows according to the index matrix *L*_2_ to obtain *P*_6_.

(21) $$\begin{array}{c} P_{51}=rot90(rot90(P_{5}))\\ P_{6}(i,j)=P_{51}(L_{2}(i,j),j) \end{array}$$

**Step 9:** Rotate *P*_6_ counterclockwise by 180° and then perform the bit-cycle operation according to *W*_2_. If *W*_2_(*i*, *j*) = 1, then *P*_61_(*i*, *j*) is shifted left by one bit. If *W*_2_(*i*, *j*) = 2, then *P*_61_(*i*, *j*) is shifted left by two bits. Similarly, if *W*_2_(*i*, *j*) = 7, then *P*_61_(*i*, *j*) is shifted left by seven bits, and finally *P*_7_ is obtained.

(22) $$\begin{array}{c} P_{61}=rot90(rot90(P_{6}))\\ P_{7}=P_{6}(bit-cycle) \end{array}$$

**Step 10:** The final ciphertext image *P*_8_ is obtained by double XOR of *P*_7_.

(23) $$P_{8}=bitxor(\mathrm{mod}(bitxor(P_{7},C)+A,256),B)$$

## 4. Decryption Process

**Figure 2:**
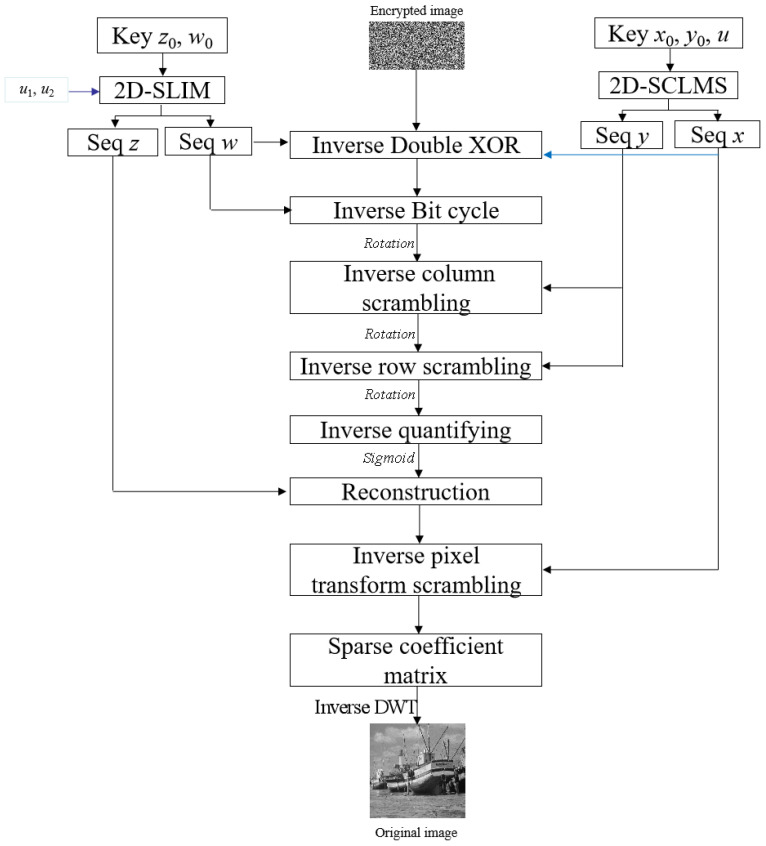
The flow chart of the decryption scenario.

The specific decryption method is demonstrated below and its flow chart is presented in Figure 2.

**Step 1:** The initial parameters are brought into the two chaotic systems. The specific method is the same as Steps 1 and 4 in Section 3.2.

**Step 2:** Perform the reverse operation of the double XOR on the ciphertext image *P*_8_ to obtain *P*_7_, then perform the inverse operation of the bit cycle and rotate 180° counterclockwise to obtain *P*_6_.

(24) $$\begin{array}{c} P_{7}=IXOR(P_{8})\\ P_{61}=P_{7}(Ibit-cycle)\\ P_{6}=rot90(rot90(P_{61})) \end{array}$$

**Step 3:** Perform the inverse scrambling operation on the rows of *P*_6_ according to the index matrix *L*_2_ and then rotate 180° counterclockwise to obtain *P*_5_.

(25) $$\begin{array}{c} P_{51}(L_{2}(i,j),j)=P_{6}(i,j)\\ P_{5}=rot90(rot90(P_{51})) \end{array}$$

**Step 4:** Perform the inverse scrambling operation on the columns of *P*_5_ according to the index matrix *L*_1_ and then rotate 180° counterclockwise to obtain *P*_4_.

(26) $$\begin{array}{c} P_{41}(i,L_{1}(i,j))=P_{5}(i,j)\\ P_{4}=rot90(rot90(P_{41})) \end{array}$$

**Step 5:** Perform inverse quantization on *P*_4_ according to the sigmoid function introduced in Section 2.2 to obtain *P*_3_.

(27) $$P_{3}=-\log(\frac{a}{P_{4}}-1)\times \frac{1}{b}+c$$

**Step 6:** Use the smooth *l*_0_ norm method to reconstruct *P*_2_.

(28) $$P_{2}=SL_{0}(P_{3},\Phi )$$

**Step 7:** Divide *P*_2_ into four blocks on average and perform inverse pixel transformation to obtain *P*_1_.

(29) $$P_{1}=IPT(P_{2})$$

**Step 8:** Perform the reverse DWT on *P*_1_ to acquire the decrypted image *P*.

(30) $$P=\Psi^{T}\times P_{1}\times \Psi$$

## 5. Simulation Experiment and Performance Analysis

Multiple experiments were conducted to prove the performance of the newly presented compressed encryption scenario. The operating system used for all experiments was Windows 10 Ultimate with AMD Ryzen 2.00 GHz CPU, 8 G RAM, and 1 TB hard disk and the operating software was MATLAB R2020a. The test selected six images with a size of 512 × 512 (“Lena”, “Cameraman”, “Cattle”, “Einstein”, “Boat” and “Couple”) and three images with a size of 256 × 256 (“Barbana”, “Lena”, “Cameraman”). 

### 5.1. Simulation Results

**Figure 3:**
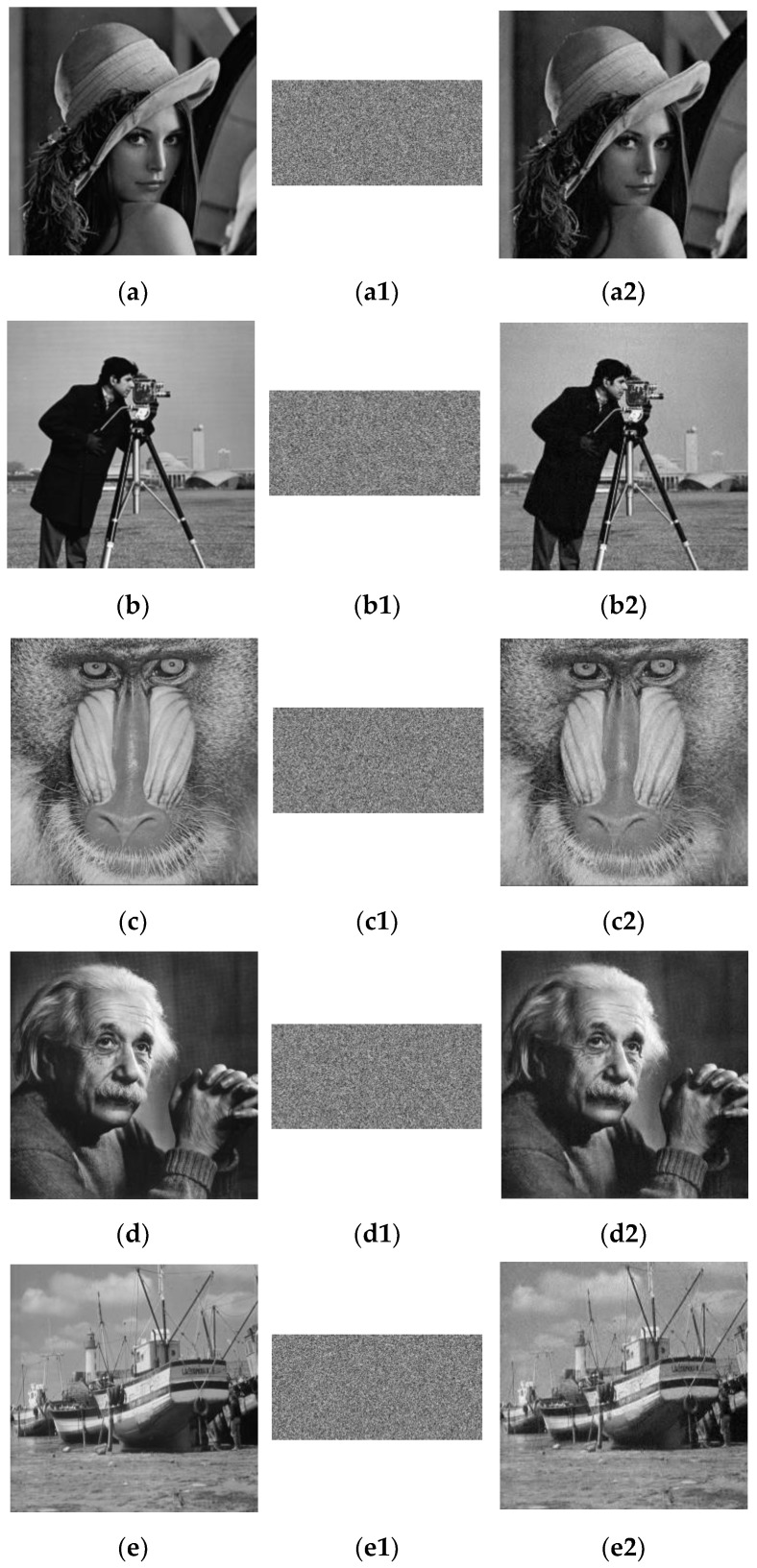

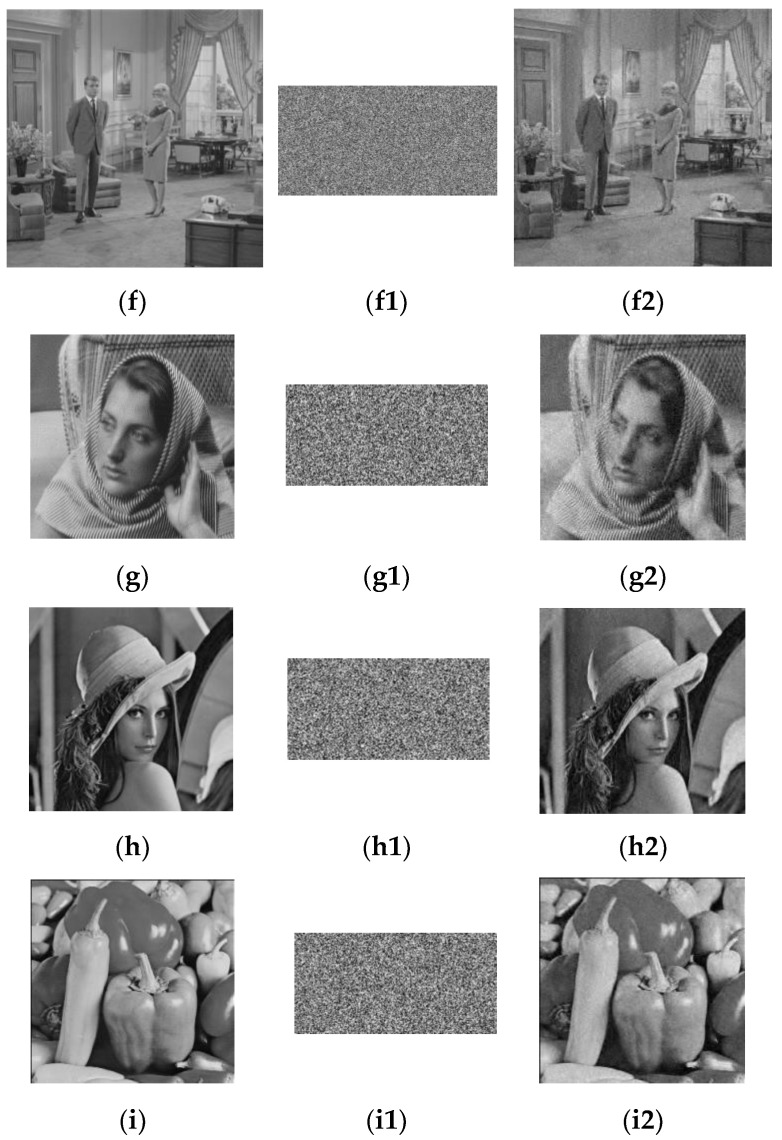
The simulation results. (**a**–**i**) Plain image; (**a1**–**i1**) encrypted image; (**a2**–**i2**) decrypted image.

Figure 3 displays the original images, compressed encrypted images, and decrypted images for all of the test images. All experiments were verified with a compression ratio of 0.5 as an example. Hereon, the original images in lines (a)–(f) are 512 × 512 and the original images in lines (g)–(i) are size of 256 × 256.

The ciphertext images were similar in noise and were smaller than the original images in Figure 3, which indicates that this scheme has a good compression and encryption effect. Furthermore, the decrypted images were of high quality and were the same size as the plaintext images, which showed that the scenario had a good reconstruction and decryption effect.

### 5.2. Compression Performance Analysis

#### 5.2.1. Peak Signal-to-Noise Ratio (PSNR)

PSNR [49] was utilized for the assessment of the compression performance. This is expressed as

(31) $$\begin{array}{c} MSE=\frac{1}{N\times N}\sum_{i=1}^{N}\sum_{j=1}^{N}{\left(X(i,j)-Y(i,j)\right)}^{2}\\ \;PSNR=10\times \log_{10}(\frac{255\times 255}{MSE}) \end{array}$$

**Figure 4:**
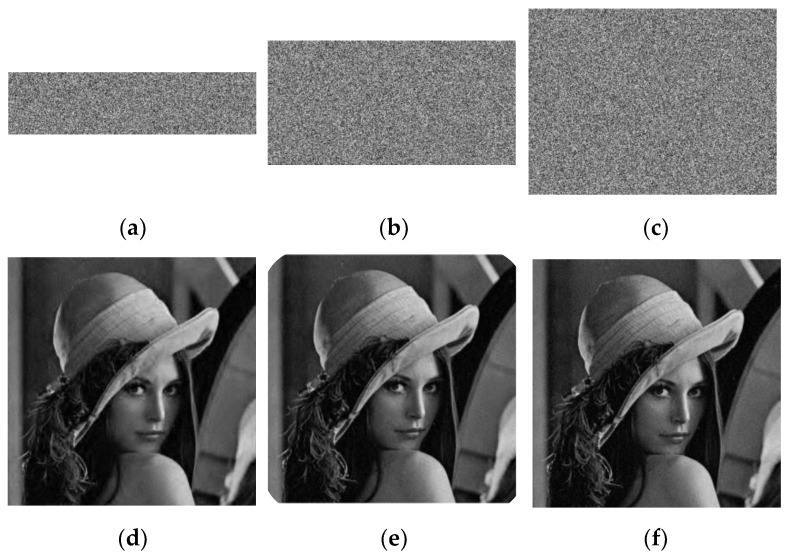
The encryption and decryption results of “Lena” with different CRs. (**a**) CR = 0.25; (**b**) CR = 0.5; (**c**) CR = 0.75; (**d**) PSNR = 33.3376 db; (**e**) PSNR = 33.9387 db; (**f**) PSNR = 32.6499 db.

In Equation (31), *X* and *Y* are the plaintext and the decrypted image, respectively. The larger the PSNR value, the better the compression performance. Figure 4 shows the simulation of “Lena” under different CRs and their corresponding PSNR values. It can be concluded that even if the CR = 0.25, the PSNR exceeded 30 db. Table 1 lists the PSNR of different images. The PSNR of the tested images exceeded 30 db, which indicates that the compression characteristic of the scenario was excellent and stable. Table 2 compares the PSNR of different compression encryption algorithms for “Lena” (256 × 256). The PSNR of our algorithm was 32.6176, which was higher than the other scenarios, which showed that the newly proposed scenario was better.

#### 5.2.2. Structural Similarity Index Measurement (SSIM)

A momentous indicator to survey the similarity of two images is SSIM, and its range is [0, 1]. The larger the SSIM [51], the greater the similarity of the two images. The expression of SSIM is

(32) $$SSIM(X,Y)=\frac{\left(2\mu_{X}\mu_{Y}+{\left(K_{1}L\right)}^{2})(2\sigma_{XY}+{\left(K_{2}L\right)}^{2}\right)}{\left(\mu_{X}^{2}+\mu_{Y}^{2}+{\left(K_{1}L\right)}^{2})(\sigma_{X}^{2}+\sigma_{Y}^{2}+{\left(K_{2}L\right)}^{2}\right)}$$

In Equation (32), *X* and *Y* are the plaintext and reconstructed image. *μ_X_* and $\sigma_{X}^{2}$ are the mean value and the variance of *X*, respectively. *μ_Y_* and $\sigma_{Y}^{2}$ are the mean value and the variance of *Y*, respectively. *σ_XY_* is the covariance of *X* and *Y*. *M* is the total number of windows. *L* = 255. *K*_1_ = 0.01, *K*_2_ = 0.03. We tested the SSIM values for multiple images, as shown in Table 3. The SSIM of the images was close to 1, which indicates that the plaintext image and reconstructed image had high similarity (i.e., the reconstruction algorithm achieved good results). Table 4 compares the SSIM of different compression encryption algorithms for “Lena” (256 × 256). The SSIM calculated by the newly proposed scenario was larger, which shows that the image reconstructed by the new scenario was more similar to the plaintext image.

### 5.3. Key Space Analysis

The key space of a scenario must be larger than 2^100^ to ensure that the algorithm is good and secure enough against brute force attacks [52].

The new algorithm has an internal key *α* and utilizes the hash-384 algorithm. Assuming that the computer has a computational precision of 10^−14^, the entire key space is 10^14^ + 2^384^, which is much larger than 2^100^. Thus, the scenario has a large key space and can resist violent attacks.

### 5.4. Key Sensitivity Analysis

A good encryption scenario is sensitive to the key, that is, even though the key changes very little, the encrypted image has a great difference.

The number of pixel change rate (NPCR) and the unified average change intensity (UACI) can be used to test the sensitivity of the scenario. These are expressed as

(33) $$\begin{array}{c} NPCR=\frac{1}{M\times N}\sum_{i=1}^{M}\sum_{j=1}^{N}\left|Sign(C_{1}(i,j)-C_{2}(i,j))\right|\\ UACI=\frac{1}{M\times N}\sum_{i=1}^{M}\sum_{j=1}^{N}\frac{\left|C_{1}(i,j)-C_{2}(i,j)\right|}{255} \end{array}$$

where *C*_1_ and *C*_2_ are two different cipher images. Table 5 lists the NPCR and UACI for multiple images.

The NPCR and UACI were close to 99.6094% and 33.4635%, respectively, which indicates that the scenario is sensitive to key.

### 5.5. Statistical Attack Analysis

#### 5.5.1. Histogram Analysis

**Figure 5:**
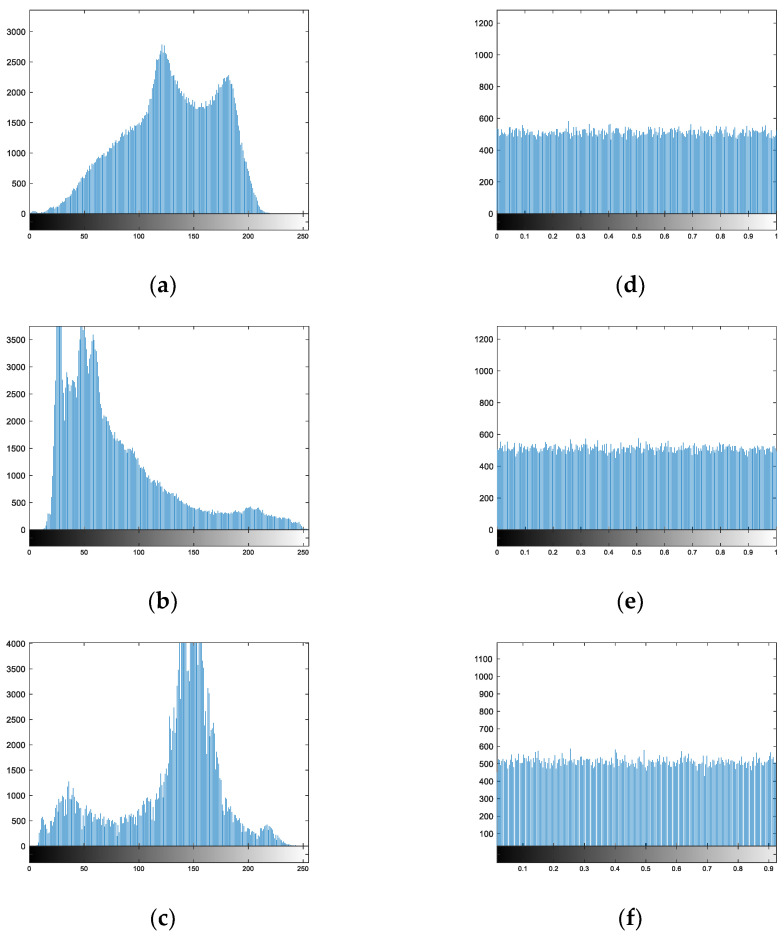
Histograms. (**a**) Plain image Cattle; (**b**) plain image Einstein; (**c**) plain image Boat; (**d**) encrypted image Cattle; (**e**) encrypted image Einstein; (**f**) encrypted image Boat.

A momentous index to appraise the performance of encryption scenarios is the histogram. Figure 5 displays the histogram of multiple plaintext images and ciphertext images.

The histograms of the plaintext images were uneven, but those of the cipher images were similar to the uniform distribution, which illustrates that the scenario resisted statistical attacks.

In addition, we utilized the histogram variance to survey the effectiveness of this algorithm.

(34) $$Var(Z)=\frac{1}{256^{2}}\sum_{i=0}^{N-1}\sum_{j=0}^{N-1}\frac{{\left(z_{i}-z_{j}\right)}^{2}}{2}$$

In Equation (34), *z_i_* and *z_j_* represent the number of pixel values corresponding to *i* and *j*. The histogram variance of the plaintext images were very large, and the maximum could reach 10^6^, while those of the ciphertext images were small, only 10^2^, and the minimum was 115.8203 in Table 6. This shows that the histogram of the ciphertext images was flatter.

Table 7 compares the histogram variance of “Lena” (256 × 256) with different algorithms. The histogram variance of the new scenario was smaller, explaining that the histogram was flatter. That is, the newly proposed algorithm was more resistant to statistical attacks.

To appraise the performance of the new scenario to resist statistical attacks, this paper utilized the chi-square [53], the expression of which is

(35) $$\chi^{2}=\sum_{i=0}^{2^{8}-1}\frac{{\left(u_{i}-u_{0}\right)}^{2}}{u_{0}}$$

In Equation (35), *u_i_* is the frequency of value *i*. *u*_0_ = *MN*/2^8^. Table 8 enumerates the chi-square results of multiple images. The values for seven images were less than 293.2478 (255 degrees of freedom and 5% confidence), which shows that this algorithm has good effects and can resist statistical attack. Table 9 compares the results of several scenarios for “Lena” (256 × 256). The chi-square value of the newly proposed encryption scenario was the smallest, which shows that this scenario was more resistant to statistical attacks.

#### 5.5.2. Correlation Analysis

The encryption scenario is to break the correlation of the original image. The evaluation index to assess the effectiveness of the scenario is the correlation coefficient, the expression of which is

(36) $$\begin{array}{c} \rho_{xy}=\frac{cov(x,y)}{\sqrt{D(x)D(y)}}\\ \mathrm{cov}(x,y)=\frac{1}{N}\sum_{i=1}^{N}\left(x_{i}-\frac{1}{N}\sum_{i=1}^{N}x_{i})(y_{i}-\frac{1}{N}\sum_{i=1}^{N}y_{i}\right)\\ D(x)=\frac{1}{N}\sum_{i=1}^{N}{\left(x_{i}-\frac{1}{N}\sum_{i=1}^{N}x_{i}\right)}^{2} \end{array}$$

In Equation (36), *x* and *y* are the image adjacent pixels. Table 10 enumerates the correlation coefficients of different original images and ciphertext images. The comparison values of “Lena” (256 × 256) with several encryption scenarios are enumerated in Table 11.

The correlation coefficients of the plaintext images were close to 1, but those of the ciphertext images were about 0 in Table 8, and that value in our scenario was smaller in Table 11, which shows that the scenario had better resistance to statistical attacks.

**Figure 6:**
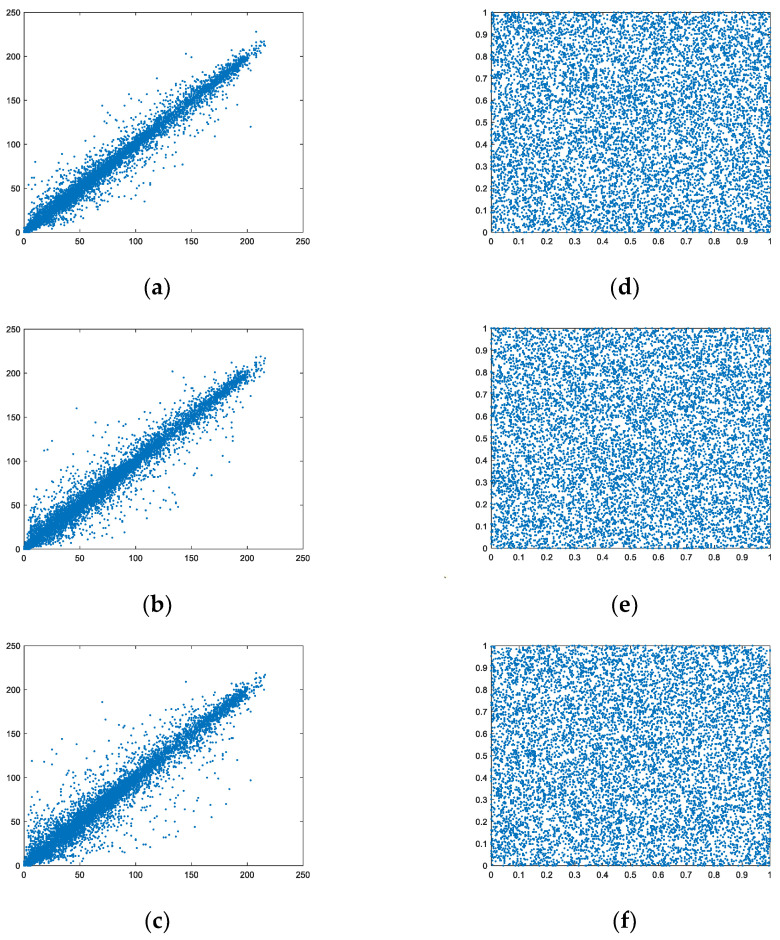
Correlation. (**a**–**c**) Horizontal, vertical, and diagonal directions of plain “Lena”; (**d**–**f**) Horizontal, vertical, and diagonal directions of encrypted “Lena”.

The correlation of “Lena” is presented in Figure 6 for clear observation. The correlation of the plaintext image in three directions was diagonal, but those of the ciphertext image were interspersed over the whole range. This shows that the encryption scenario effectively abated the correlation.

#### 5.5.3. Information Entropy (IE)

The quota to assess the overall randomness of images is the IE and its expression is

(37) $$H(s)=-\sum_{i=0}^{M}p(s_{i})\log_{2}p(s_{i})$$

In Equation (37), *M* = 255. *p*(*s_i_*) is the probability of *s_i_*. The closer the IE is to 8, the better the algorithm [54].

Table 12 lists the IE of several original and ciphertext images. Table 13 lists the comparison value of “Lena” (256 × 256) using different algorithms.

The IE of the ciphertext image was extremely close to 8 in Table 12. The IE of the newly encryption algorithm was higher than the other algorithms in Table 13. Therefore, this algorithm had very good results. The encrypted image had stronger randomness and was resistant to statistical attacks.

#### 5.5.4. Local Information Entropy (LIE)

A momentous metric for analyzing the randomness of the local image is the LIE. Some non-overlapping image blocks are randomly selected and the LIE can be obtained by calculating the IE of each block and then taking the average value. The expression is

(38) $$\bar{LH_{k,T_{B}}}(P)=\sum_{i=1}^{k}\frac{H(S_{i})}{k}$$

where *H*(*S_i_*) is the IE of sub-block *S_i_*. Let *k* = 30, *T_B_* = 1936 for calculation. When the confidence level is 0.05, the range of LIE is [7.901901305, 7.903037329] [32]. Table 14 lists the LIE of different images (512 × 512). The LIE of all test images passed the experiment, which shows that the local image had good randomness.

### 5.6. Differential Attack Analysis

An excellent encryption scenario is sensitive to the plaintext image, in other words, even though the original image has very small changes, the encrypted image can be completely different.

The NPCR and UACI are indicators used to measure whether the algorithm can resist differential attacks. When the NPCR > NPCR*_α_, the NPCR passes the test. When the UACI is between [UACI*^−^_α_, UACI*^+^_α_], the UACI passes the test [55]. The NPCR and UACI statistical tests are shown in Table 15 and Table 16.

The NPCR and UACI of all test images were very close to the ideal values, and all passed the NPCR and UACI tests. Therefore, the algorithm could effectively resist differential attacks.

### 5.7. NIST SP 800-22 Analysis

The NIST SP 800-22 statistical test suite is published by the National Institute of Standards and Technology for testing sequences for randomness [56]. Therefore, we set the confidence level to 0.01 to evaluate the randomness of the ciphertext image. The results are listed in Table 17. All data passed the test, indicating that the ciphertext image had good randomness.

### 5.8. Time Complexity

Time complexity is an important quantitative criterion to evaluate the feasibility of an encryption algorithm, and it requires the algorithm to be easy to execute. If the running time of the algorithm is too long, it does not meet the requirements of real-time performance. This paper tested the encryption time of multiple images, which are presented in Table 18. The time of all 256 × 256 images was less than 1 s, and the time of 512 × 512 images was less than 3 s, which greatly proves that the algorithm is real-time.

### 5.9. Anti-Noise Attack Analysis

**Figure 7:**
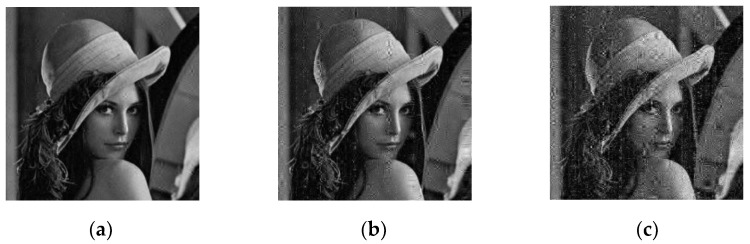
Noise attack: (**a**) 0.005% noise; (**b**) 0.05% noise; (**c**) 0.1% noise.

As it is subject to various noise interference during transmission, an excellent encryption scenario should resist noise attacks. The salt and pepper noise is tested at intensities of 0.005%, 0.05%, and 0.1% in “Lena”, as shown in Figure 7.

Even though the added noise intensity was 0.1%, the cipher image could be decrypted and information could be viewed. This shows that the scenario resisted noise attacks.

In order to measure the anti-noise ability of the encryption algorithm more accurately, this paper tested the PSNR. For three different noise intensities, their corresponding PSNR are presented in Table 19. When the noise intensity was 0.005%, the PSNR was 33.2311, even if the noise intensity increased to 0.1%, the PSNR was greater than 29, which shows that the algorithm had a strong resistance to noise.

## 6. Discussion

The encryption algorithm based on the chaotic system and compressed sensing proposed in this paper could resist various attacks, and had security and timeliness. However, it also has certain limitations. The measurement matrix is generated by the universal method, that is, the chaotic sequence generated by the chaotic system constitutes the measurement matrix. We should conduct further research in the future to make better use of the chaotic characteristics of the chaotic system to construct a better measurement matrix to make the compression and encryption more convenient and obtain better compression and encryption effects.

## 7. Conclusions

The paper proposed a new image compression and encryption scenario based on CS and two chaotic maps. The pixel transform operation was performed before the compressed sensing first, which is beneficial to increase the image reconstruction quality. In the quantization process, we made full use of the performance of the sigmoid function to quantize the matrix to the interval [0, 255]. In the scrambling process, we combined rotation with row and column scrambling, which tremendously reduced the correlation. Finally, the cipher image was created by double XOR after the bit-cycle operation.

After a series of tests and experimental analysis, the new scenario had a huge key space and was sensitive to keys. In addition, various experiments against statistical analysis attacks were carried out in this paper such as histograms and their statistical analysis, information entropy, correlation, and local information entropy. The information entropy was very close to 8, and the correlation coefficient was close to 0. Subsequently, the algorithm was also resistant to differential attacks, brute force attacks, and noise attacks. All of the test images were close to the standard values of the NPCR and UACI and passed the statistical analysis test, and their PSNR exceeded 29 for 0.1% intensity noise. The bit sequence of the ciphertext image passed the NIST randomness test.

The significance of this paper was to combine the two chaotic systems with compressed sensing, which can not only fully utilize the practicability of chaos theory for image encryption, but can also compress ciphertext images to meet the needs of the transmission bandwidth. The encryption algorithm proposed in this paper is not only resistant to various attacks, but also has real-time performance and is a secure encryption scheme.

In the future, we should focus on the further combination of the chaotic system and compressed sensing and its application in medicine or larger fields.

## Figures and Tables

**Table 1:** The PSNR (db) of the proposed algorithm for different images.

Image	PSNR
Lena	33.9387
Cameraman	32.3925
Boat	31.1828
Couple	31.1743
Einstein	32.4941
Peppers	32.4268

**Table 2:** The PSNR (db) comparison for several schemes.

Image	Ref. [31]	Ref. [48]	Ref. [50]	Ours
Lena (256 × 256)	30.71	29.23	31.2302	32.6176

**Table 3:** The SSIM for different images.

Image	SSIM
Lena	0.9001
Cameraman	0.8323
Boat	0.8447
Couple	0.8313
Einstein	0.8704
Cattle	0.8021
Peppers	0.7082

**Table 4:** The SSIM comparison for different algorithms.

Image	Ref. [48]	Ref. [50]	Ours
Lena (256 × 256)	0.7129	0.6475	0.7337

**Table 5:** The key sensitivity.

Image	Key	
	NPCR	UACI
Lena	99.5911%	33.4233%
Einstein	99.6094%	33.5026%
Couple	99.6117%	33.4125%
Cattle	99.6017%	33.3956%
Boat	99.6056%	33.4358%
Cameraman	99.5834%	33.5923%
Peppers	99.6307%	33.5688%
Barbana	99.5880%	33.5250%

**Table 6:** The histogram variance of multiple images.

Image	Original Image	Encrypted Image
Lena	1.0827 × 10^6^	461.9766
Couple	1.1955 × 10^6^	515.0078
Cameraman	1.6741 × 10^6^	584.1484
Boat	1.5359 × 10^6^	558.7188
Einstein	1.1987 × 10^6^	455.4297
Cattle	7.5077 × 10^5^	466.6719
Barbana	6.0765 × 10^5^	136.4609
Peppers	3.6777 × 10^4^	115.8203

**Table 7:** The histogram variance comparison of “Lena” (256 × 256) using different algorithms.

Plain Image	Ref. [37]	Ref. [50]	Ours
3.0665 × 10^4^	181.7109	121.4063	105.6328

**Table 8:** The chi-square values.

Image	Lena	Couple	Einstein	Cattle	Boat	Peppers	Barbana
Chi-square	230.9883	257.5039	227.7148	233.3359	279.3594	231.6406	272.9219
	270,681.8	298,865.2	299,672.0	187,692.2	383,969.7	106,323.0	1,248,061.3

**Table 9:** The chi-square of “Lena” (256 × 256) using different algorithms.

Plain Image	Ref. [37]	Ref. [50]	Ours
30,665.7	253.3125	242.8125	211.2656

**Table 10:** The correlation coefficients for different images.

Image	Horizontal	Vertical	Diagonal
Lena	0.9840	0.9835	0.9717
	−0.0032	−0.0037	−0.00085
Cameraman	0.9853	0.9870	0.9765
	−0.00014	−0.0017	0.0030
Boat	0.9833	0.9727	0.9617
	−0.0015	0.0058	−0.0018
Couple	0.9624	0.9650	0.9386
	0.0037	0.00045	0.0014
Einstein	0.9687	0.9644	0.9548
	−0.0014	−0.00043	−0.00021
Cattle	0.8573	0.9103	0.8423
	−0.0057	−0.0016	−0.0015
Peppers	0.9490	0.9452	0.9039
	−0.0048	−0.0021	−0.0014
Barbana	0.9056	0.7542	0.7158
	−0.00066	0.0057	0.0018

**Table 11:** The correlation coefficients of “Lena” (256 × 256) using different schemes.

Direction	Horizontal	Vertical	Diagonal
Plain image	0.9746	0.9651	0.9539
Ref. [31]	0.0009	−0.0062	−0.0087
Ref. [37]	−0.0160	−0.0044	−0.0052
Ref. [48]	−0.0015	0.0041	0.0069
Ref. [50]	0.0076	−0.0066	−0.0035
Ours	0.0012	−0.0041	0.0032

**Table 12:** The IE of several images.

Image	Original Image	Encryption Image
Lena	7.3920	7.9987
Couple	7.2010	7.9986
Cameraman	7.0480	7.9987
Boat	7.1914	7.9985
Einstein	7.2655	7.9987
Cattle	7.3579	7.9987
Peppers	7.5327	7.9949
Barbana	5.0030	7.9940

**Table 13:** The information entropy of “Lena” (256 × 256) using different algorithms.

Plain Image	Ref. [37]	Ref. [48]	Ref. [50]	Ours
7.5683	7.9944	7.9935	7.9946	7.9954

**Table 14:** The LIE (512 × 512).

Image	LIE	Result
Lena	7.902316286	Pass
Cameraman	7.902787296	Pass
Boat	7.902113612	Pass
Cattle	7.902520981	Pass
Einstein	7.902336151	Pass
Couple	7.902842150	Pass

**Table 15:** The NPCR statistical test.

**Image**	**NPCR**	**Theoretical NPCR Critical Value**		
512 × 512		N*_0.001_ = 99.5717%	N*_0.01_ = 99.5810%	N*_0.05_ = 99.5893%
		0.001-level	0.01-level	0.05-level
Lena	99.5941%	Pass	Pass	Pass
Einstein	99.6460%	Pass	Pass	Pass
Couple	99.5987%	Pass	Pass	Pass
Cattle	99.6185%	Pass	Pass	Pass
Boat	99.6185%	Pass	Pass	Pass
Cameraman	99.6048%	Pass	Pass	Pass
**Image**	**NPCR**	**Theoretical NPCR Critical Value**		
256 × 256		N*_0.001_ = 99.5341%	N*_0.01_ = 99.5527%	N*_0.05_ = 99.5693%
		0.001-level	0.01-level	0.05-level
Lena	996368%	Pass	Pass	Pass
Barbana	996368%	Pass	Pass	Pass
Peppers	996154%	Pass	Pass	Pass

**Table 16:** The UACI statistical test.

**Image**	**UACI**	**Theoretical UACI Critical Value**		
512 × 512		N*^−^_0.001_ = 33.3115%	N*^−^_0.01_ = 33.3445%	N*^−^_0.05_ = 33.3730%
		N*^+^_0.001_ = 33.6156%	N*^+^_0.01_ = 33.5826%	N*^+^_0.05_ = 33.5541%
		0.001-level	0.01-level	0.05-level
Lena	33.4078%	Pass	Pass	Pass
Einstein	33.5236%	Pass	Pass	Pass
Couple	33.4989%	Pass	Pass	Pass
Cattle	33.5140%	Pass	Pass	Pass
Boat	33.4173%	Pass	Pass	Pass
Cameraman	33.4373%	Pass	Pass	Pass
**Image**	**UACI**	**Theoretical UACI Critical Value**		
256 × 256		N*^−^_0.001_ = 33.1594%	N*^−^_0.01_ = 33.2255%	N*^−^_0.05_ = 33.2824%
		N*^+^_0.001_ = 33.7677%	N*^+^_0.01_ = 33.7016%	N*^+^_0.05_ = 33.6447%
		0.001-level	0.01-level	0.05-level
Lena	33.4428%	Pass	Pass	Pass
Barbana	33.4929%	Pass	Pass	Pass
Peppers	33.5082%	Pass	Pass	Pass

**Table 17:** The NIST SP 800-22 test.

Test Items	*p*-Value	Results
Frequency test	0.332829	Pass
Block frequency test	0.589821	Pass
Cusum-forward test	0.577516	Pass
Cusum-reverse test	0.201550	Pass
Runs test	0.315933	Pass
Longest run test	0.471291	Pass
Rank test	0.452825	Pass
FFT test	0.510298	Pass
Non-overlapping template test	0.510816	Pass
Overlapping template test	0.387884	Pass
Universal test	0.545638	Pass
Approximate entropy test	0.463226	Pass
Random-excursions test (x = −1)	0.159822	Pass
Random-excursions variant test (x = 1)	0.124450	Pass
Serial1 test	0.550327	Pass
Serial2 test	0.584547	Pass
Linear complexity test	0.403982	Pass

**Table 18:** The encryption runtime (Unit: s).

Image	Lena	Couple	Einstein	Cattle	Boat	Lena	Peppers	Barbana
Time	2.8783	2.9142	2.8466	2.9652	2.8521	0.9598	0.9463	0.9421

**Table 19:** The PSNR test for noise resistance.

Noise	0.005%	0.05%	0.1%
PSNR	33.2311	29.5916	29.1613

## Data Availability

Data sharing not applicable.
